# Sonography only in emergency? The black and the white

**DOI:** 10.1186/1824-7288-40-S1-A61

**Published:** 2014-08-11

**Authors:** Paolo Adamoli

**Affiliations:** 1 Pediatric Department, Moriggia Pelascini General Hospital, Gravedona ed Uniti (Como), Italy

## 

The ultrasound transducer generates ultrasounds. It emits them, collects the return echo and allows the ultrasound system to re-create an image that (few people) know how to interpret.

The probe and the ultrasound system work together by measuring the liquid component of a tissue, as ultrasounds propagate easily trough the water.

On sonography imaging liquids appear black because they are “anechoic”. It means that the ultrasound wave goes through them without emitting any return echo .

Tissues containing a lot of water appear dark , or “hypoechoic” , because the larger part of the ultrasound waves go through them while a small part of them is reflected by the tissues and returns to transducer.

Otherwise less hydrated tissues appear lighter, or “hyperechoic”, because they reflect the largest amount of ultrasound.

On sonography, a solid tissue appears white with a black shadow below, as it reflects all the ultrasounds.

The air does not reflect ultrasound but scatters them back toward the probe. The return echo signals are interpreted as dispersion artifacts.

With these simple concepts, it is easier to interpret many ultrasound images.

The search of liquid effusions, anechoic, in the context of serous cavities after a trauma, in well known as FAST sequence and its evolution EFAST .

This sequence is a versatile and valuable tool, also available for those physicians inexperienced in sonography .

The FAST sequence is feasible anywhere (point of care) and without mobilizing the inpatient (bedside) or during CPR .

The versatility of this sequence has allowed many physicians without specific training or background to approach clinical sonography.

Ultrasound focused lung sequence (LUS) was proposed for respiratory disease, very frequent in pediatrics.

The presence of tipical artifacts allow to detect a regular air intake in lung parenchyma near next to the chest wall. Their absence is a sure sign of respiratory disease.

A question naturally arises: why should we use ultrasound just in urgency?

There is no unambiguous answer, but after the last decade, the US scanner is becoming a valuable tool to further investigate the physical examination, especially in emergency department.

In Table 1 it summarizes that a basic sonography could be the chest examination in order to evaluate the air intake Simultaneously, a further application could be addressed to the research of anechoic areas and/or hyperechoic pathological patterns of abdomen, not just after trauma.

**Table 1:** 

Pathological aspects in thorax ecography	
1	Pleural effusion

2	Bronchopneumonia thickening

3	Pneumothorax

4	Complex pathological aspects (thymic ectopia, neonatal respiratory distress)

Pathological aspects in abdominal ecography with anechoic areas (serous effusions)	

1	Ascitis

2	haemoperitoneum

3	hydronephrosis

4	hydrocele

5	Complex pathological aspects (renal cysts, splenic cysts, hepatic cysts, ovarian cysts, cystic duplications)

Pathologic abdominal sets with echogenic areas and acoustic shadow	

1	Cholelithiasis

2	Hepatic calcification

3	Nephrolithiasis

4	Appendicolith

During the last year an interesting application was referred to the detection of skull fractures.

This easy and bedside procedure presents good sensitivity (88%) and high specificity (97%).

All of these procedures appear useful tools for the optimization of waiting times of diagnostic imaging in emergency (golden hour!) and for reduction of ionizing radiations use in pediatric patients.

